# Knowledge, attitudes and decision regret: a longitudinal survey study of participants offered genome sequencing in the 100,000 Genomes Project

**DOI:** 10.1038/s41431-023-01470-1

**Published:** 2023-10-03

**Authors:** Michelle Peter, Jennifer Hammond, Saskia C. Sanderson, Jana Gurasashvili, Amy Hunter, Beverly Searle, Christine Patch, Lyn S. Chitty, Melissa Hill, Celine Lewis

**Affiliations:** 1 NHS North Thames Genomic Laboratory Hub, Great Ormond Street Hospital for Children NHS Foundation Trust, London, UK; 2 Genetics and Genomic Medicine, UCL Great Ormond Street Institute of Child Health, London, UK; 3 Genetic Alliance UK, London, UK; 4 Unique - Rare Chromosome Disorder Support Group, Oxted, UK; 5 Engagement and Society, Wellcome Connecting Science, Wellcome Genome Campus, Hinxton, CB10 1RQ UK; 6 Population, Policy and Practice Department, UCL Great Ormond Street Institute of Child Health, London, UK

**Keywords:** Genomics, Social sciences

## Abstract

We used cross-sectional surveys to compare the knowledge, attitudes, and decision regret of participants who had consented for genome sequencing (GS) for rare disease diagnosis in the 100,000 Genomes Project (100kGP) across two timepoints (at the time of consenting for GS (T1) and 12–18 months later (T2)). At T1, participants (*n* = 504) completed a survey that included measures of general knowledge of GS (“Knowledge of Genome Sequencing” (KOGS)), specific knowledge of GS and attitudes towards GS (“General attitudes” and “Specific attitudes”). At T2, participants (*n* = 296) completed these same assessments (apart from the specific knowledge scale) together with an assessment of decision regret towards GS (“Decisional Regret Scale”). At 12–18 months after consenting for GS, participants’ basic knowledge of GS had remained stable. General knowledge of GS varied across topics; concepts underlying more general information about genetics were better understood than the technical details of genomic testing. Attitudes towards GS at T2 were generally positive, and feelings towards GS (both positive and negative) remained unchanged. However, those who were more positive about the test at the outset had greater specific knowledge (as opposed to general knowledge) of GS. Finally, although the majority of participants indicated feeling little regret towards undergoing GS, those with low positive attitude and high negative attitude about GS at T1 reported greater decision regret at T2. Careful assessment of patient knowledge about and attitudes towards GS at the time of offering testing is crucial for supporting informed decision making and mitigating later regret.

## Introduction

Genome sequencing (GS) has recently been introduced into mainstream healthcare in England. Offered through the National Health Service England’s (NHSE) Genomic Medicine Service, GS is now being used in clinical settings to aid the diagnosis of rare and inherited diseases in children and adults. There is enormous potential for GS to increase the number of diagnoses made, bringing benefits that will include a clearer prognosis, information about recurrence risk, more accurate treatments, the chance to take part in research projects, and opportunities to obtain support [1–3].

The 100,000 Genomes Project (100kGP) was a hybrid clinical and research project designed to prepare for implementation of GS in the NHS. The NHS genomic medicine service (GMS) was launched in England in October 2018 and GS for patients with selected cancers and undiagnosed rare genetic diseases was introduced into routine care through the GMS in 2021 [4, 5]. In the 100kGP, patients with some cancers or one of 190 rare and inherited diseases, together with their parents and relatives, were recruited between 2015 and 2018. 100kGP participants were asked to consent to receive main findings from GS and to contribute their data for research [6]. An initial discussion with potential participants about taking part in the 100kGP was undertaken by NHS clinicians from both mainstream and genetics backgrounds. Potential participants were given a participant information leaflet. The professionals who took consent for the 100kGP came from a variety of backgrounds, including genetic counselling, research or other post graduate training. All were trained to take consent, including taking the online course developed for the 100kGP. Participants were consented to the project in either conversations conducted in person or by phone. GS results were returned to 100kGP participants by their referring NHS clinicians from either mainstream or genetics backgrounds with any follow-up care delivered via routine clinical pathways.

Several studies, including our own, have made use of the wealth of data amassed from the 100kGP, with many exploring the consent processes and participants’ motivations for undergoing GS [2, 7–8]. However, as more people are faced with the complex decision-making surrounding GS, there is a need for further research that examines people’s understanding of, attitudes towards and long-term satisfaction with undergoing this type of genetic test. Research in this area is already underway. For instance, we previously reported the experiences of decision regret and the psychological impacts for 100kGP participants’ who had received a GS result from the project. We explored these concepts from two perspectives: 1) those who had received a diagnosis from GS versus those who had not and, 2) those who had received a result as a patient versus those who had received a result as a parent for their child. Although we observed no differences in levels of regret between parents and patients, or between those with a diagnosis and those without, our findings indicated that parents experienced higher levels of distress and uncertainty after receiving their GS compared to patients [9] indicating that parents may need additional emotional support during post-test counselling. Outside of the context of the 100kGP, research has indicated that patients are mostly positive about GS, report minimal regret or harm from undergoing GS [10, 11] and are satisfied with their clinician’s communication when returning GS results [12].

These insights are useful for our understanding but, as NHSE’s Genomic Medicine Service becomes more established, a more comprehensive picture of how patients’ knowledge and feelings about GS develop over time is needed. This information will be essential for clinicians who may need to consider tailoring discussions according to an individual’s level of understanding, and to policy makers, who could use the findings to develop guidelines that will assist clinicians in providing adequate patient support during the pre- and post-test counselling process.

In our overarching mixed-methods study, we used cross-sectional surveys distributed to 100kGP participants at two time points (following consent of GS (T1) and 12–18 months later (T2)) and qualitative interviews with people who had received results at T2. We have previously reported participants’ knowledge and attitudes to taking part in the 100kGP at T1 [13], and for those who had received a result at T2, their experiences of decision regret and psychological impacts at T2 [9]. In this report, we assessed the data from all participants who completed the T1 and T2 surveys and used it to:

1. explore the knowledge, attitudes, and decision regret of 100kGP participants at least 12 months after consenting for GS for rare disease diagnosis (T2),
2. compare whether knowledge and attitude changed over time (comparison of T1 and T2), and
3. investigate the relationship between knowledge and attitude at the point of consenting for GS (T1) and decision regret at least a year on (T2).

## Methods

### Ethical approval

Ethical approval was obtained from the NHS Research Ethics Committee West Midlands (15/WM/0258).

### Study design

Two surveys were distributed to participants in the rare disease arm of the 100kGP: one following the offer of GS (T1) and another 12–18 months later (T2).

### Survey content

The development and dissemination of the T1 and T2 surveys have been described elsewhere [13]. General knowledge of GS was measured through an assessment of “Knowledge of Genome Sequencing (KOGS)” [14], and specific knowledge about GS was assessed at T1 only using a scale comprising 28 items that could be True, False or Don’t Know (e.g., *The results from whole-genome sequencing will definitely show the cause of the rare condition in your family*). To measure attitudes, participants completed a scale that assessed general attitudes to GS (e.g., *For me (and my child), having whole GS isbeneficial/harmful*) and specific attitudes to GS (e.g., *I feel that taking part could help my child get a diagnosis*). Decision regret was measured using the Decisional Regret Scale (DRS) [15]. Table 1 describes how the assessments were scored.

**Table 1:** Scoring for assessments used to measure knowledge, attitudes and regret towards GS.

	Measure	Scale	Scoring
**Knowledge and understanding**	(General) Knowledge of Genome Sequencing (KOGS)	Nine items. Tick box (3 options): True (1), False (0), Not sure (0)	Range = 0–9. Items 1, 3, 4, and 9 are TRUE; scored as 1. Items 2, 5, 6, 7, 8 are FALSE; scored as 0. Total score obtained by summing all item scores. 0 = low knowledge, 9 = high knowledge. A higher score indicates higher level of knowledge.
	Specific knowledge of GS^a^	28 items. Tick box (3 options): True (1), False (0), Don’t know (0)	Range = 0–28. Items 1, 4, 6, 10, 12, 13, 14, 16, 17, 18, 20, 23, 25, 26, 27, 28 are TRUE; scored as 1. Items 2, 3, 5, 7, 8, 9, 11, 15, 19, 21, 22, 24 are FALSE; scored as 0. Total score obtained by summing all item scores. 0 = low knowledge, 28 = high knowledge. A higher score indicates higher level of knowledge.
**Attitudes**	General attitudes towards GS	Four items. 5-point Likert scale: Harmful / Unimportant / A bad thing / Unhelpful (1) to Beneficial / Important / A good thing / Helpful (5).	Range = 4–20. 1 = negative attitude, 5 = positive attitude. Total score obtained by summing item scores. A higher score indicates a positive attitude.
	Specific attitudes towards GS	14 items. 5-point Likert scale: Strongly disagree (1) to Strongly Agree (5).	Range = 7–35. Seven items relate to perceived benefits (positive attitude) and seven to perceived concerns (negative attitude). Scores obtained by summing item scores from each subscale. Higher scores on the benefits items denote higher positive attitude, and higher scores on the concers items denotes higher negative attitude.
**Decisions**	Decisional regret scale (O’Connor 1996 (modified 2003))	Five items. 5-point Likert scale: Strongly agree (1) to Strongly disagree (5).	Range = 0–100. Items 2 and 4 were reverse coded so that for all items a higher number indicates greater regret. Scores were converted to a 0–100 scale by subtracting 1 from each item and then multiplying by 25. A final score was obtained by summing item scores and then averaging across items. Higher scores indicate greater decision regret.

*GS* Genome sequencing.

^a^assessed at T1 only.

### Participants and recruitment

Participants were recruited from six London hospitals that were part of two Genomic Medicine Centres involved in recruiting probands and their relatives into the 100kGP. Participants included adult patients, parents of children, and relatives of patients all with a rare disease undergoing GS. The T1 survey was conducted between 1st July 2017 and 30th September 2018. Approximately 12–18 months after returning T1, respondents with complete contact details (*n* = 504) were invited to complete either a paper or online version of the T2 survey via SurveyMonkey between 1st March 2019 and 16th October 2020.

### Data analysis

Correlations and comparative analyses were conducted to identify relationships between relevant demographic variables at T1 (education and age since these could influence knowledge and attitudes), relationships between testing variables at T1, and to detect changes over time (between T1 and T2). Spearman’s correlation was used to test the association between individuals’ ratings at T1 and T2, and Wilcoxon signed rank tests were used to assess group differences in ratings between T1 and T2. Depending on the type of variables included, decision regret scores were analysed as either continuous data or classified into three categories that have been used elsewhere in the literature [16] where 0 = no regret; 5–25 = mild regret; and > = 30 = moderate to strong regret. ANOVA and chi-squared tests were used for comparative analysis of categorical variables. All analyses were conducted using R 4.0.2 [17].

## Results

At T1, 504 surveys were received, and 296 at T2 (58.7% response rate) (see Table 2 for participant characteristics). Of the T2 surveys, 77 were from participants who reported receiving a GS result. Details about the type of result received by these participants are reported elsewhere [9]. All other participants (*n* = 219) still had pending results at T2.

**Table 2:** Characteristics of T2 survey respondents.

Characteristic		*N* (%)
Participant type	Patient	141 (48%)
	Parent	123 (42%)
	Other relative	25 (8%)
	*Missing*	7 (2%)
Gender	Female	175 (59%)
	Male	114 (39%)
	*Missing*	7 (2%)
Age, years	Mean (SD), range	48.0 (14.0), 16–79
Currently employed	Yes	194 (66%)
	No	99 (33%)
	Do not wish to answer	2 (1%)
	*Missing*	1 (0.3%)
Education	No qualification	14 (5%)
	GCSE or O level	50 (17%)
	GCE, A-level or similar	22 (7%)
	Vocational e.g. BTEC	67 (23%)
	Bachelors degree	81 (27%)
	Masters degree	38 (13%)
	PhD, MD, JD	13 (4%)
	*Missing*	11 (4%)
Ethnicity	White or White British	248 (84%)
	Asian or Asian British	21 (7%)
	Black or Black British	4 (1%)
	Mixed	7 (2%)
	Other ethnic group	8 (3%)
	*Missing*	8 (3%)
Religious faith	None	104 (35%)
	Christian	155 (52%)
	Muslim	12 (4%)
	Hindu	5 (2%)
	Jewish	1 (0.3%)
	Buddhist	1 (0.3%)
	Sikh	1 (0.3%)
	Other	13 (4%)
	*Missing*	4 (1%)
Religiosity (How religious are you?)	Not at all	145 (49%)
	Somewhat	115 (39%)
	Very	25 (8%)
	*Missing*	11 (4%)
No. of children (range 0–9)	0	73 (25%)
	1	52 (18%)
	2	102 (35%)
	3	43 (15%)
	4 or more	20 (7%)
	*Missing*	6 (2%)
Age of child/relative patient, years	Mean (SD), range	13.4 (13.6), 0–74

### Knowledge of GS

#### General knowledge of Genome Sequencing (KOGS)

At T2, the scale had moderate internal consistency (α = 0.67). Overall, participants (*n* = 291) indicated moderate general knowledge of GS at T2: the mean score was 5.14 (*SD* = 2.12, median = 5.00, range = 0–9), where 0=low and 9=high general understanding of GS. Some items, however, were subject to more variation than others. For instance, knowledge was mixed when responding to the item, *there are uncertainties about what a person’s genome can tell them:* 65% *(n* = 186*)* correctly answered this as true, but 28% (*n* = 80) were unsure, and 8% (*n* = 20) incorrectly answered this as false. People were also unsure as to whether *GS involves looking at around half of the DNA in a genome:* 41.8% (*n* = 121) correctly stated that this is false, but around half (48.1%; *n* = 139) did not know. General knowledge of GS at T1 varied by education level [F(6) = 7.89, *p* < 0.001], with post-hoc Tukey tests revealing significantly higher scores for those with a postgraduate or undergraduate degree (at *p* < 0.05). There was no relationship between general knowledge at T1 and age [*rho* = −0.01, S = 21684995, *p* = 0.818]. Comparing scores between T1 and T2 showed that knowledge remained stable across individuals [*rho* = 0.50, S = 2.06e + 06, *p* < 0.001] and did not differ as a group over time [*Z* = 1.49, *p* = 0.136].

#### Specific knowledge of genome sequencing

Specific knowledge of GS was measured at T1 only. The scale had moderate internal consistency (α = 0.60) and participants (*n* = 504) showed evidence of moderate specific knowledge: the mean score was 15.32 (*SD* = 2.00, median = 15.00, range = 7–25), where 0 = low and 28 = high specific knowledge of GS. As with general knowledge of GS, some concepts were better understood than others. For instance, most people (*n* = 494; 98%) correctly answered that *Having whole-genome sequencing done might help other children in the future*, and 82% (*n* = 411) understood that *Finding the genetic cause of your child’s condition could have implications for other family members*. However, a fifth (*n* = 101; 20%) incorrectly believed that *The results from whole-genome sequencing will definitely provide a diagnosis for you/your child* and a third (*n* = 171; 33%) incorrectly answered “False” to the statement *You may not receive any informative results about your child’s condition from whole-genome sequencing*. No relationship was found between specific knowledge at T1 and age [*rho* = 0.0009, S = 21317209, *p* = 0.983] and specific knowledge did not differ by education [F(6) = 0.73, *p* = 0.623].

### Attitudes towards GS

#### General attitudes towards GS

At T2, the scale had good internal consistency (α = 0.82) and attitudes were generally positive: the majority strongly agreed that GS was beneficial (69.4%; *n* = 204), important (65.9%; *n* = 193), a good thing (72.8%; *n* = 214), and helpful (67.2%; *n* = 197). There was no relationship between general attitude at T1 and age [*rho* = −0.008, S = 21563783, *p* = 0.850], or wiith general knowledge of GS at T1 [*rho* = 0.06, S = 20583579, *p* = 0.192], or specific knowledge of GS at T1 [*rho* = 0.05, S = 20498092, *p* = 0.255]. Participants felt positively about GS at both time points: attitudes were stable across individuals over time [*rho* = 0.41, S = 2.36e + 06, *p* < 0.001] and there was no difference, overall, in general attitude between T1 and T2 [Z = 0.397, *p* = 0.691) (Table 3).

**Table 3:** General and specific attitudes towards genome sequencing at T1 and T2.

	Total score		Sig.
	T1	T2	
**General attitude (range 4–20)**			
Mean (SD)	18.28 (2.61)	18.29 (2.44)	Z = 0.397, *p* = 0.69
Median	20	20	
Range	10–20	10–20	
**Specific attitude (range (7–35)**			
*Positive attitude*			
Mean (SD)	30.19 (3.32)	29.79 (3.93)	Z = 1.19, *p* = 0.23
Median	30	30	
Range	22–35	7–35	
*Negative attitude*			
Mean (SD)	17.21 (5.51)	17.24 (5.86)	Z = 0.32, *p* = 0.75
Median	16	17	
Range	7–34	7–35	

#### Specific attitudes towards GS

At T2, internal consistency was good (α = 0.77). People saw benefits to GS and had few concerns. There was no relationship between positive attitude at T1 and age [*rho* = −0.005, S = 21563783, *p* = 0.917] nor between negative attitudes at T1 and age [*rho* = −0.05, S = 22463786, *p* = 0.296]. There was also no link between positive attitude at T1 and general knowledge at T1 [*rho* = −0.04, S = 22951114, *p* = 0.319] nor between negative attitudes at T1 and general knowledge at T1 [*rho* = −0.08, S = 23748032, *p* = 0.070]. Notably, there was an association between positive attitude at T1 and specific knowledge at T1 [*rho* = 0.20, S = 17269216, *p* < 0.001], indicating that those with greater specific knowledge about GS at the outset saw more benefits to the test. No link, however, was found between negative attitudes at T1 and specific knowledge at T1 [*rho* = 0.009, S = 21529527, *p* = 0.843]. Positive attitudes [*rho* = 0.36, S = 4.113e–10, *p* < 0.001] and negative attitudes [*rho* = 0.62, S = 2.2e–16, *p* < 0.001] remained stable across individuals between T1 and T2, and there was no difference in positive [Z = 1.19, *p* = 0.23] or negative attitude [Z = 0.32, *p* = 0.75] overall between T1 and T2.

### Decisions about GS

#### Decision regret

Decision regret (DR) was measured at T2 only. Cronbach’s alpha revealed good internal consistency (*α* = 0.90). Across all participants (*n* = 296), the mean DR score was 12.26 (*SD* = 14.41, median = 5, range = 0–80) which, given the maximum possible score of 100, shows that regret was low. Viewing the data in terms of discrete categories corroborated these findings and showed that 39 people (13.2%) had high levels of regret. No differences in DR were identified between those who had received a GS result and those who had not [χ^2^ (4) = 0.60, *p* = 0.963]. No relationship between general knowledge of GS (KOGS) at T1 and DR at T2 was found and this did not differ between those who had received a GS result and those who had not [Z = 0.649, *p* = 0.258]. Similarly, no relationship between specific knowledge of GS at T1 and DR at T2 was found, and this too did not differ between those who had received a GS result and those who had not [Z = 1.627, *p* = 0.052] However, attitudes towards GS at T1 were related to DR at T2: A negative relationship between General attitude at T1 and DR at T2 [*rho* = −0.28, S = 1.154e–06, *p* < 0.001] showed that people with a lower positive attitude at the outset of undergoing GS, felt greater regret 12–18 months later. This relationship did not differ between those who had received a GS result compared to those who had not [Z = *−*0.486, *p* = 0.313]. A similar relationship was found between Specific attitude at T1 and DR at T2: positive attitude at T1 was negatively related to DR [*rho* = −0.22, S = 0.00013, *p* < 0.001] and negative attitude at T1 was positively related to DR [*rho* = 0.28, S = 8.318e–07, *p* < 0.001], indicating that those who saw fewer benefits and who had greater concerns about GS at the outset had greater regret 12–18 months later. As with general attitudes towards GS, the relationship between specific attitudes towards GS did not differ amongst those who had received a result compared to those who had not [positive attitude: Z = *−*0.546, *p* = 0.292; negative attitude: Z = 0.241, *p* = 0.405].

## Discussion

In this study, we used cross-sectional surveys to compare the knowledge, attitudes, and decision regret of 100kGP participants at the time of consenting for GS and at least 12 months later.

Like other work in which public attitudes towards GS have been shown to be favourable [18, 19] our study showed that, in general, people felt positively about GS with most reporting it to be beneficial. Notably, we showed that attitudes towards GS remained stable over time, with both positive and negative feelings towards GS remaining unchanged for individuals between T1 and T2.

In line with other research [20, 21], our study also revealed that, 12–18 months after consenting to GS, participants felt little decision regret regardless of whether a result had been received—though this must be considered in light of the fact that only a small proportion of participants had reported receiving a result at the time of completing the T2 survey. Nonetheless, an interesting nuance to our results was that, whilst low overall, regret was highest amongst those who were the least positive and those who had the greatest concerns about GS at the time of consent (T1). This finding has important implications for clinical practice—namely, that GS should be delivered by clinicians who are skilled at ascertaining individual patient attitudes and concerns. Time should be spent exploring patients’ feelings towards GS to understand any reservations they may have, as well as their expectations about the test. To do this, clinicians could ask patients open ended questions about their feelings towards GS and whether they have any concerns. It may also help to explore their motivations for accepting or declining testing and their previous testing experience.

It is also important to examine whether patients’ attitudes towards testing are not a result of misinformation or misunderstanding about GS. Furthermore, for all patients—but especially those with a negative or ambivalent attitude, it should be made clear that not having the test is an option and that they could chose to delay the decision and potentially undergo GS at another time. Now that GS has shifted from being offered as part of a research project to being offered in a clinical care setting in England, further research with patients undergoing GS in the NHS Genomic Medicine Service is necessary to assess whether attitudes to GS continue to be positive and decision regret remains low. This is particularly important since the resources available within a research setting may not be comparable to those within a national health system.

Our work also identified that participants had basic general knowledge of GS and that this remained stable over time. We found that questions relating to concepts underlying more general information about genetics tended to be better known by most, whereas there was greater uncertainty around the more specific details of genomics and genomic testing. This pattern of moderate genetics knowledge and low knowledge of GS techniques has been shown elsewhere [22, 23]. However, the level of knowledge that is needed to support decision making is an open question. Some have proposed that, because of the complexity of genomic information and the broad range of possible results from GS, the goal of pre-test counselling should be that patients make “appropriately informed” rather than “fully informed” decisions [24]. Our work, brought to light an interesting finding that speaks to this proposition. Though no direct link between knowledge and decision regret was observed in our study, we noted an interrelation between these two concepts and participants’ attitudes: those with a better understanding of the specific details of GS viewed the test more positively, and those who viewed the test more positively had lower decision regret later on. Worthy of note, is that this relationship was only observed when measuring knowledge using the specific knowledge scale and not the KOGS. A likely reason for this is that the KOGS assesses general knowledge of GS, including items that focus on the technicalities of GS technology and on genetic and genomic literacy more broadly (e.g., *A person’s genome is the complete set of cells in their body)*. Whilst the specific knowledge scale also measures understanding of these concepts (e.g., *In order for whole-genome sequencing to be done, DNA is extracted from your blood sample)*, it is a more sensitive measure because it also includes items that explore people’s expectations about GS (e.g., *You may not receive any informative results about your child’s condition from whole-genome sequencing*). Based on these findings, we tentatively suggest that a person’s specific knowledge about GS could be used as a possible predictor of their attitudes towards it. In support of this view, we found that topics conceptualised by the specific knowledge scale were less well understood (such as the likelihood of receiving a diagnosis from GS and the potential clinical utility of GS results) and should be made clear to patients. Having a better understanding could help patients to form more realistic expectations about GS and, by association, support the formation of attitudes towards GS that matches their testing decisions.

The challenges that patient understanding of GS brings to the informed consent process have been highlighted in other studies, including having to adapt the consent conversation to varying levels of genomic literacy and managing patient expectations about the scope of the test [25]. Our findings fit with previous work that highlighted the question of how important knowledge of the technical minutiae of genomic testing is for informed choice when patients are faced with complex decisions surrounding GS [13]. We speculate, however, that by supporting patients’ understanding about the more specific details about GS, such as the benefits and limitations of the test, clinicians can impact attitudes towards GS which, in turn, could mitigate against later regret.

Offering information about GS in alternative formats is one way to support patient knowledge—the potential benefits of doing so have been highlighted, with research showing that educational animated videos are effective in increasing knowledge about whole genome sequencing [26] and are positively endorsed by viewers [27]. Developing easily accessible, multi-format information about GS could facilitate the patient-clinician conversation and provide an additional resource to help patients consolidate what has been discussed during counselling. Another option could be the adoption of a two-step consent conversation model as proposed by Johnson and colleagues [28] who found that this structured approach improved genetic knowledge for parents whose children were offered clinical genomic testing. Providing patients offered GS with the opportunity to discuss the test and its possible implications over two sessions would afford patients the time to raise concerns they had not considered during their first consent conversation. Furthermore, repetition of details about the test (which families have identified as a model clinician behaviour) could help to facilitate absorption of information.

## Strengths and limitations

An important limitation of this study is that only a small proportion of participants had reported receiving a result at the time of completing the T2 survey. There may also be response bias towards participants with strong feelings about GS and participants without a result who may have been more inclined to engage with the research team to express disappointment. In addition, as this was a cross-sectional study, responses are limited to the attitudes and experiences at a given point in time.

## Conclusions

As GS becomes established within routine NHS care, studies must continue to assess the support required by patients being offered this test. This is particularly important because the resources available within a research setting like the 100kGP may not be comparable to those within a national health system. Our study has highlighted the need for a service in which clinicians are able to identify patients’ level of understanding and their expectations about the utility of GS and subsequently tailor their discussions so that patients are given the best chance to feel positively about their testing decision. By adopting this model from the outset, clinicians can support patients to avoid making decisions they later regret. Our work has also shown the need for further research exploring the factors that contribute to attitude formation about GS and identifying what support would help to improve psychosocial outcomes of GS. Finally, learning why it is that some patients feel regret about undergoing GS could help us enhance pre-test counselling guidelines that better support their needs when offered this test.

## Data Availability

Anonymised data underlying the results is accessible through the UCL Data Repository [10.5522/04/21931677]. Data will be made available under the terms of the Creative Commons Attribution 4.0 (CC BY 4.0).
